# *In vivo *tissue uptake of intravenously injected water soluble all-*trans *β-carotene used as a food colorant

**DOI:** 10.1186/1475-2891-8-56

**Published:** 2009-12-01

**Authors:** Tomoko T Yamanushi, Midori I Torii, Najma Janjua, Hideaki Kabuto

**Affiliations:** 1 Kagawa Prefectural College of Health Sciences, 281-1 Hara, Mure-cho, Takamatsu City, Kagawa 761-0123, Japan

## Abstract

Water soluble β-carotene (WS-BC) is a carotenoid form that has been developed as a food colorant. WS-BC is known to contain 10% of all-*trans *β-carotene (AT-BC). The aim of the present study was to investigate *in vivo *tissue uptake of AT-BC after the administration of WS-BC into rats. Seven-week-old male rats were administered 20 mg of WS-BC dissolved in saline by intravenous injection into the tail vein. At 0, 6, 24, 72, 120 and 168 hours (n = 7/time), blood was drawn and liver, lungs, adrenal glands, kidneys and testes were dissected. The levels of AT-BC in the plasma and dissected tissues were quantified with HPLC. After intravenous administration, AT-BC level in plasma first increased up to 6 h and returned to normal at 72 h. In the testes, the AT-BC level first increased up to 24 h and then did not decrease but was retained up to 168 h. In the other tissues, the level first increased up to 6 h and then decreased from 6 to 120 or 168 h but did not return to normal. The accumulation of WS-BC in testes but not in the other 5 tissues examined may suggest that AT-BC was excreted or metabolized in these tissues but not in testes. Although WS-BC is commonly used as a food colorant, its effects on body tissues are still not clarified. Results of the present study suggest that further investigations are required to elucidate effects of WS-BC on various body tissues.

## Findings

Carotenoids are one of the main groups of coloring substances in nature [1-3]. The advantages to add carotenoids as food colorants are: high tinctorial potency, safety, stability, compatibility and availability. As color conveys a concept of freshness and wholesomeness by an ingrained color-taste expectancy relationship, the technical challenge of the food industry has been to create suitable application forms of carotenoids for food coloring needs [1-4]. Several application forms of the carotenoids have been developed for coloring both fat-based (margarine, cheese, butter, etc.) and water-based (juice, beverages, etc.) foods [4]. Because carotenoids are fat soluble, three approaches were used to overcome this disadvantage: 1) reduction in crystal size; 2) preparation of emulsions in liquid and beadlet forms; and 3) development of colloidal preparations [4]. Since various application forms of β-carotene have been developed and are most widely used as food colorants, people intake β-carotene easily in their daily life. β-Carotene is one of the provitamin A carotenoids, which is cleaved to retinal, followed by its conversion to retinyl ester within the small intestine [5-8]. Vitamin A and its analogs (retinoids) are needed to maintain normal growth and development, immunity, reproduction and other essential physiologic processes [8-10]. Besides the provitamin A activity, β-carotene has other important biological functions such as quenching of singlet oxygen, interrupting peroxidation, reducing the free radicals and so on [3,6]. Many epidemiological studies over a long period have reported a negative relationship between β-carotene intake and chronic disease [reviewed in [6,11]]. However, two large recent trials found that pharmacological levels of β-carotene increased lung cancer incidence and deaths in smokers [12,13] and asbestos workers [13]. A larger trial with healthy American men, however, found no effect of β-carotene on several types of malignant neoplasms except an increased risk for thyroid and bladder cancer [14]. These contradictory reports [6,11-14] suggest a possible dual response of β-carotene, whereby it promotes health when taken at dietary levels, but may have adverse effects when taken at higher doses [15]. The tissue distribution of β-carotene is still not clearly defined [15]. In the present study, we aimed to examine rat *in vivo *tissue uptake of all-*trans *β-carotene (AT-BC). The studies of β-carotene distribution are difficult because several factors, such as the solubility conditions and *in vivo *digestion, influence its absorption. Therefore, the choice of solvent and the method of β-carotene administration have been controversial [16]. In the present study, we used water soluble β-carotene (WS-BC) which has been commonly used as one of the application forms of food colorants. The WS-BC is considered to overcome the β-carotene insolubility and its absorption difficulties.

Dry β-carotene beadlets (Trade name: Dry β-Carotene 10% Cold Water-Soluble) were kindly donated by Hoffmann La Roche Japan, Co., Ltd. (Tokyo, Japan). The beadlets contained 10% of AT-BC. In addition, the beadlets consisted of vehicle (starch, gelatin, sucrose and plant oil) and antioxidants (vitamin E and vitamin C). In this paper, the dry β-carotene beadlets refer to as water soluble β-carotene (WS-BC). The reagents used were purchased from Wako Pure Chemical Industries Inc. (Osaka, Japan). The solvents for high performance liquid chromatography (HPLC) were purchased from Nacalai Tesque Inc. (Kyoto, Japan). Sprague-Dawley male rats (7 weeks old, body weight 210-230 g) were purchased from CLEA Japan, Inc. (Tokyo, Japan) and housed under constant temperature on a 12-hour light/dark cycle. Before the intravenous injection of WS-BC, rats were fed standard laboratory diet (CE-2, CLEA Japan, Inc., Tokyo, Japan) and given access to tap water *ad libitum *for 5 days. To make WS-BC solution, 20 mg of WS-BC was dissolved in saline, and 1 ml of this solution was injected into the tail vein of the rat. Thus, the actual amount of AT-BC administered into the rat was 2 mg. The intravenous injection of WS-BC was performed as described previously [16]. At 0, 6, 24, 72, 120 and 168 hours after the intravenous administration of WS-BC, blood was drawn from the abdominal vein and centrifuged to obtain plasma. Rats were sacrificed, and liver, lungs, adrenal glands, kidneys and testes were removed and frozen in liquid nitrogen. For each time point, 7 rats were used. The removed organs and plasma were kept at -80°C until further analysis. All experimental protocols were conducted in accordance with Japanese Act on Welfare and Management of Animals (Act No. 105 of October 1, 1973). The quantification of AT-BC in the tissues was performed using HPLC as described before [16]. Data were presented as means ± SD. Statistical analyses were carried out using Origin (Microcal Software Inc., USA) or Excel (Microsoft, USA) software. Differences were considered significant at the level of p < 0.05.

**Figure 1:**
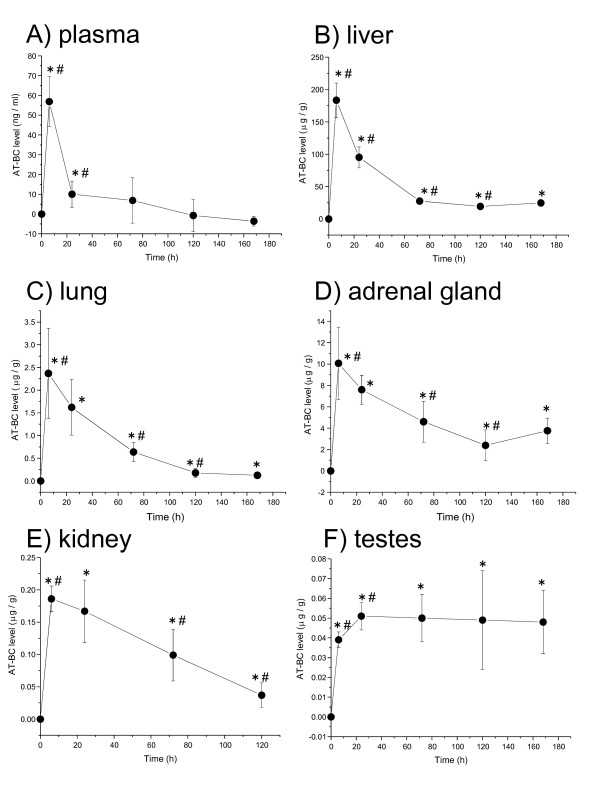
**Time dependent changes in AT-BC levels in 6 tissues after WS-BC intravenous administration in rat**. Values plotted are mean ± SD (n = 7/time). For each tissue, individual AT-BC values at each time point were adjusted by subtracting the respective mean values at 0 hour, and mean ± SD were then calculated. * significantly different as compared with AT-BC level at 0 h. # significantly different as compared with AT-BC level at the preceding time point.

The mean (± SD) AT-BC levels in the tissues were examined at 6 time points after the intravenous administration (Figure 1). The AT-BC levels in all tissues increased up to 6 h (Figure 1). From this, it may be assumed that the AT-BC circulated in the blood stream and was distributed to these tissues within 6 h. In plasma, the AT-BC level decreased rapidly after 6 h and the level at 72 h was not significantly different from that at 0 h (Figure 1A). This may suggest that all the AT-BC administered in plasma was distributed to other body tissues or excreted from blood by 72 h. The AT-BC level in the liver decreased over a period of 6 h to 120 h (Figure 1B). In the lung, adrenal gland and kidney, the AT-BC levels decreased gradually from 6 h to 120 or 168 h (Figure 1C, D and 1E). This may indicate that the AT-BC was excreted from these tissues or was metabolized to the possible metabolites of AT-BC, retinoids and carotenoid isomers [5-8,15]. The AT-BC levels in lung, adrenal gland and kidney at 120 and/or 168 h were still significantly different from those at 0 h (Figure 1C, D and 1E). This observation could suggest that some amount of AT-BC was still retained at 120 or 168 h in these tissues. In the testes, AT-BC level first increased up to 24 h and then did not decrease but was retained up to 168 h (Figure 1F).

In the present study, *in vivo *tissue uptake of AT-BC was examined after the intravenous administration of WS-BC into rats. The *in vivo *emulsifying conditions have been shown to affect β-carotene absorption [15,17]. In our pilot experiments, when the rats were fed a refined diet containing WS-BC, the level of AT-BC in serum, liver and lung showed a wide variation between animals (data not shown). This may suggest that *in vivo *tissue uptake of AT-BC is affected by individual absorption conditions of WS-BC. Therefore, oral administration of WS-BC is considered difficult to examine *in vivo *tissue uptake. Furthermore, in our previous work involving intravenous administration of the emulsified AT-BC crystals in solvent, high levels of AT-BC accumulated in the lung [16]. This was suggested to be due to the trapping of the solvent used for dissolving AT-BC [16]. To overcome these problems of absorption and insolubility of AT-BC, in the present study, WS-BC was dissolved in saline and administered intravenously. However, like our previous study [16], in some tissues including lung, accumulation of a large amount of AT-BC was not found in the present study. This would suggest that the WS-BC was not trapped by the solvent in the present study whereas it was in the previous study [16].

The antioxidant properties of β-carotene are strictly dependent on oxygen partial pressure (OPP) [18,19]. *In vitro *experiments at different OPP have demonstrated ambiguous behavior of β-carotene [18]. At OPP less than the oxygen pressure in normal air, β-carotene behaved as an antioxidant whereas at higher values, it was found to lose its antioxidant activity and actually showed a pro-oxidant effect [18]. A number of reports have now confirmed this phenomenon in purified systems [20], microsomes [21], cell lines [22] and bacteria [23]. In addition, after β-carotene administration to rats, cytochrome P450 isoforms were induced and reactive oxygen species (ROS) were increased in kidney, lung, intestine and liver, with liver being the most affected tissue [19]. From these findings, it was suggested that β-carotene may have two contradicting behaviors, antioxidant and pro-oxidant [19]. Oxidative stress plays a major contributory role in pathogenesis of many generative and chronic diseases [19]. Many epidemiological studies have been reported which show a negative relationship between dietary β-carotene intake and chronic disease [6,11]. On the other hand, recent intervention trials suggest that β-carotene supplementation may promote health when taken at dietary levels but have adverse effects when taken in higher amounts [11,15]. The conflicting behavior of β-carotene may explain why the contradictory results were obtained in different studies.

In the present study, the observation of accumulation of AT-BC over a period of 168 h after the intravenous administration of WS-BC (Figure 1F) suggests that under certain conditions, testes may have the ability to store AT-BC for several days. In an immunohistochemical study [7], human β-carotene 15,15'-mono-oxygenease (BCO1), which is involved in the symmetrical cleavage of β-carotene into two retinal molecules, was detected in steroidogenic cells in testis, ovary, and adrenal gland [24]. The level of mRNA of carotene cleavage enzyme (CCE), which cleaves provitamin A carotenoid to retinol, was found to be highest in testis among 4 tissues examined including the small intestine [8]. From these observations, it was suggested that in testis, β-carotene could act as a local source of retinoids, which have been shown to be important during proliferation, differentiation, and maturation of germinal cells [7,8,24]. In male rats treated with fenvalerate [25] or cadmium [26], administration of β-carotene was reported to ameliorate the induced toxicity in the testis. Moreover, β-carotene administration increased semen quality [25]. Overall, from these findings, it appears that β-carotene may be essential for the function of testes. Because β-carotene is commonly used commercially to color food, people intake it easily in their daily life. Since this provitamin has an ambiguous behavior as becoming antioxidant or pro-oxidant depending on its partial oxygen pressure, results of the present study suggest that further investigations are required to elucidate its effect on body tissues under various physiological conditions.

## List of abbreviations

WS-BC: water soluble β-carotene; AT-BC: all-*trans *β-carotene; HPLC: high performance liquid chromatography; OPP: oxygen partial pressure.

## Competing interests

The authors declare that they have no competing interests.

## Authors' contributions

TTY was the main author of the manuscript and contributed to the design of the study, preparation of protocols, carrying out of the experiments, interpretation of data and preparation of the manuscript. MIT carried out all the experiments, and contributed to the statistical analyses, interpretation of data and preparation of the manuscript. NJ contributed to interpretation of data and writing, editing and proof reading of the manuscript. HK contributed to interpretation of data and preparation of the manuscript. All authors read and approved the final manuscript.
